# Ultralong C100 Mycolic Acids Support the Assignment of *Segniliparus* as a New Bacterial Genus

**DOI:** 10.1371/journal.pone.0039017

**Published:** 2012-06-14

**Authors:** Sunhee Hong, Tan-Yun Cheng, Emilie Layre, Lindsay Sweet, David C. Young, James E. Posey, W. Ray Butler, D. Branch Moody

**Affiliations:** 1 Division of Rheumatology, Immunology and Allergy, Brigham and Women’s Hospital and Harvard Medical School, Boston, Massachusetts, United States of America; 2 Division of Tuberculosis Elimination, National Center for HIV, STD and Tuberculosis Prevention, Centers for Disease Control and Prevention, Atlanta, Georgia, United States of America; French National Centre for Scientific Research - Université de Toulouse, France

## Abstract

Mycolic acid-producing bacteria isolated from the respiratory tract of human and non-human mammals were recently assigned as a distinct genus, *Segniliparus,* because they diverge from rhodococci and mycobacteria in genetic and chemical features. Using high accuracy mass spectrometry, we determined the chemical composition of 65 homologous mycolic acids in two *Segniliparus* species and separately analyzed the three subclasses to measure relative chain length, number and stereochemistry of unsaturations and cyclopropyl groups within each class. Whereas mycobacterial mycolate subclasses are distinguished from one another by R groups on the meromycolate chain, *Segniliparus* species synthesize solely non-oxygenated α-mycolates with high levels of *cis* unsaturation. Unexpectedly *Segniliparus* α-mycolates diverge into three subclasses based on large differences in carbon chain length with one bacterial culture producing mycolates that range from C58 to C100. Both the overall chain length (C100) and the chain length diversity (C42) are larger than previously seen for mycolic acid-producing organisms and provide direct chemical evidence for assignment of *Segniliparus* as a distinct genus. Yet, electron microscopy shows that the long and diverse mycolates pack into a typical appearing membrane. Therefore, these new and unexpected extremes of mycolic acid chemical structure raise questions about the modes of mycolic acid packing and folding into a membrane.

## Introduction

The *Actinomycetales* order includes bacteria that are markedly divergent in their infectious potential. They range from deadly pathogens, like *Mycobacterium tuberculosis*, to facultative pathogens and those living solely in the environment [1]. Biomedical interest in species in the intermediate range of pathogenicity has greatly increased in recent years. Facultative pathogens such as corynebacteria and rhodococci are not highly pathogenic in healthy humans but cause disease in immunocompromised human hosts infected with HIV, patients treated with chronic immunosuppressive drugs or in cystic fibrosis patients. Some evidence indicates that chronically colonizing bacteria may trigger immune responses that were previously considered autoimmune diseases [2]. Finally, comparisons of highly virulent and facultative pathogens provide insight into mechanisms of virulence.

Genera in the suborder *Corynebacterineae* contain mycolic acids, which form into an outer membrane in the cell envelope and confer characteristic properties of bacteria in this suborder. Mycolic acids are α-alkyl-β-hydroxy fatty acids, which are divided into subgroups based on functional groups in the meromycolate chain, including oxygen modifications, unsaturations and cyclopropyl groups. The variability in carbon chain length, as well as the number and nature of meromycolate substitutions, are the result of specific fatty acyl synthases, cyclopropanation enzymes, and other multi-enzyme complexes [3], [4], [5]. Genera containing these mycolic acids have been termed mycolata.

The different chemical functional groups and carbon chain lengths of the mycolate structure are valuable as chemotaxonomic markers. For example, species in the genus *Mycobacterium* have the longest reported mycolic acid chains and so can be distinguished from other actinobacteria based on this property. Mycobacterial mycolates are typically C60–88 and display the greatest chemical functionality within the meromycolate chains. The other mycolata demonstrate a varied degree of unsaturation and a diverse carbon chain length, including *Corynebacterium* (C22–36), *Rhodococcus* (C34–54), *Nocardia* (C50–62), *Gordonia* (C54–66) and *Tsukamurella* (C64–74) [3]. Mycobacterial mycolic acids assemble into the mycolate outer membrane (MOM), with the majority of the mycolic acids covalently bound by an ester linkage to the nonreducing termini of D-arabinan of the arabinogalactan. The covalently bound mycolic acids intermix with noncovalently bound mycolic acids, mycolyl glycolipids and other hydrophobic lipids [6]. Mycolic acid in the cell envelope layers confers characteristic acid-fast staining properties and creates a relatively impermeable barrier, which promotes intracellular survival and contributes to the relative drug resistance of these bacteria [7], [8], [9].

Recently, rapidly growing, acid-fast staining bacteria generated scientific interest because they were isolated from sputa or bronchial washings of human patients, suggesting natural colonization or respiratory infection, yet they were untypable by standard criteria [10]. Phylogenetic analysis of 16S rRNA gene suggested a genetic relationship with *Rhodococcus* species. However, reversed phase HPLC of mycolates showed late elution, suggesting that these untypable species produce mycolic acids that were unusually hydrophobic and likely of longer chain length than those normally found in *Rhodococcus* species. Whereas most rapidly growing mycolata stain weakly acid fast, the high intensity of the acid-fast stain was surprising, a second finding that suggested possibly unique mycolate structures in these unusual bacteria [9]. Polyphasic characterization studies revealed divergent properties that contrast with known mycolata, leading to the assignment of a new genus with two species, *Segniliparus rotundus* and *Segniliparus rugosus*. These findings from human isolates, along with isolation of a strain from a seal, suggest that *Segniliparus rugosus* may represent an emerging infectious disease associated with environmental water sources or cystic fibrosis [11], [12], [13].

Here we describe high accuracy mass spectrometry and nuclear magnetic resonance to establish the chemical composition and structure of 65 molecular species belonging to three classes of *Segniliparus* mycolates. The overall length of mycolic acids, which reaches C100, is distinctly atypical of rapid growing mycolata. To our knowledge, these lipids represent the longest mycolic acids known and are among the longest lipids known in cell biology. Discovery of new extremes in mycolate chemical diversity support new models for the spatial distribution and folding of mycolic acids in the *Segniliparus* cell envelope.

## Materials and Methods

### Bacterial Strains

Strains used in this study include *Segniliparus rotundus* strain CDC 1076^T^ ( = ATCC BAA-972^T^ = CIP 108378^T^), *Segniliparus rugosus* sp. nov. type strain CDC 945^T^ ( = ATCC BAA-974^T^ = CIP 108380^T^) [10], and *Mycobacterium tuberculosis* strain H37Rv ( = ATCC 27294^T^).

### Growth Conditions

Bacteria were grown in sterile filtered (0.2 µm) Middlebrook 7H9 medium containing 0.2% glycerol (w/v) and 10% (v/v) Middlebrook ADC enrichment. Cultures were incubated at 37°C for 4 days after which cells were collected by centrifugation at 3,500 rpm, 20°C for 15 min. Cells were washed three times, resuspended in 10 ml fresh growth medium, and cellular debris was allowed to settle. Five ml of suspended fine growth culture was transferred to 45 ml medium and incubated for 4 days, after which time cells were collected by centrifugation and sterilized with gamma irradiation at 2.0 megarad.

### Scanning and Transmission Electron Microscopy (SEM and TEM)

For scanning electron microscopy, cells were fixed with 5% glutaraldehyde in cacodylate buffer (0.067 mol, pH 6.2) for 4 hours at room temperature. Suspensions were filtered using a 0.1 um polycarbonate filter (Porectics Corporation, Livermore, CA). Filters were dehydrated in a graded series of ethanol washes and immersed in Hexamethyldisilazane (Polysciences, Warrington, PA) overnight at room temperature. Finished specimens were mounted on aluminum stubs with silver paint, coated with 30 nM of gold and observed using a Philips XL Environmental Scanning Electron Microscope (FEI Company, Portland, OR). For transmission electron microscopy, cells were placed into formalin for 1 hr, washed 3 times for 2 min each in 0.1 M phosphate buffer and transferred to a 1% solution of OsO4 for 1 hour. Cells were washed with water and dehydrated in a graded series of ethanol washes, collected and placed into embedding molds with 100% resin solution. The resin was polymerized at 50°C for 8 hours, cooled and cut into sections on a copper grid. Uranyl acetate and lead citrate were added, dried and the specimens were observed with a Phillips Tecnai™ G^2^ Electron Microscope [14].

### Isolation, Methylation and Thin-layer Chromatography (TLC) of Mycolic Acids

Total mycolates were isolated by saponification as previously described, using new, clean glassware and HPLC grade solvents [15]. Briefly, the wet cell pellet (50 mg) was transferred into 2 mL potassium hydroxide (25%, w/v) in 1∶1 methanol: water (v/v) was added and heated in at 100°C for 3 hrs. After cooling, 2 ml chloroform was added, followed by 1.5 ml hydrochloric acid in water (50%, v/v), and the fatty acids were separated by centrifugation, dried under nitrogen, treated with 100 ul potassium bicarbonate (2%, w/v) in 1∶1 (v/v) methanol and water and centrifuged. After removing supernatant, all free fatty acids were dried and re-dissolved in 1 ml tetrabutylammonium hydrogen sulfate (3.39%, w/v) in water followed by 1 ml methylene chloride and 100 ul iodomethane (Sigma) at room temperature for 30 min, centrifuged at 1000 rpm for 5 min, treated with 1 ml 1 M HCl. The lower phase was collected, dried down under nitrogen and analyzed by TLC (Silica Gel 60, Macherey-Nagel) using 5 developments with 94∶6 (v/v) petroleum ether: ethyl acetate [16], [17] and developed with 8% (v/v) phosphoric acid and 3% (w/v) cupric acetate and charring. Purification of mycolate subclasses was achieved by preparative TLC, after which bands were scraped, extracted with chloroform and used for further analyses.

### Liquid-chromatography-electrospray Ionization *-*Time of Flight Mass Spectrometry (LC-ESI-Tof-MS) and Collision-induced Dissociation Mass Spectrometry (CID-MS) Analysis of Mycolic Acids

Total and preparative MAMEs were analyzed by LC-ESI0 MS using an Agilent Technologies 6520 Accurate-Mass Q-Tof (Agilent, Santa Clara, CA) connected to an Agilent 1200 series HPLC system using a Varian Monochrom 3 µm×150 mm×2 mm diol column. The samples were suspended at 1 mg/ml in solvent A [Hexane:Isopropanol 70∶30 (v:v), 0.02% formic acid, 0.01% ammonium hydroxide] and filtered. Ten µg of lipid was injected, and the column was eluted at 0.15 mL/min with a binary gradient from 0% to 100% solvent B [isopropanol: methanol 70∶30 (v:v), 0.02% formic acid, 0.01% ammonium hydroxide]: 0–10 min, 0% B; 17–22 min, 50% B; 30–35 min, 100% B; 40–44 min, 0% B, followed by additional 6 min 0% B post-run as described in detail [18]. The ionization source was maintained at 325°C with a drying nitrogen gas flow of 5 L/min, a nebulizer pressure of 30 psi and a capillary voltage of 3000 V. Spectra were collected in the positive-ion mode from 100 to 3000 m/z at 1 spectra/s. Internal calibration was performed using proprietary standards of 121.050873 and 922.009798 *a.m.u*. Collision induced dissociation (CID)-MS experiments were performed on a LXQ 2 dimensional ion-trap mass spectrometer (Thermo Scientific) equipped with an electrospray ionization (ESI) source. For the positive-ion mode analysis, 1-D TLC purified samples were dissolved in chloroform/methanol (1∶1, v/v) at ∼10 µg/ml. Five µl of sample were mixed with a solution of 1 mM sodium acetate in methanol and loaded into a nanospray tip and analyzed by electrospray ionization-MS. Bacterial pellets of *S. rugosus* and *S. rotundus* were extracted with chloroform, which were subsequently diluted with methanol to a final concentration of ∼10 µg/ml, loaded into a nanospray tip (5 µl), and analyzed with a spray voltage and capillary temperature were set to 0.8 kV and 200°C. The collision energy was 28∼35% of maximum and the trapping of product ions were carried out with a *q* value of 0.2 or 0.25.

### Proton Nuclear Magnetic Resonance (1H-NMR) Analysis

1H-NMR spectra were recorded on a Barnett Instrument 500 MHz at room temperature in 100 µg/ml deuterated chloroform in 200×5 mm 535-pp Wilmad NMR tubes with tetramethyl silane as a proton reference. Chemical shifts are expressed in ppm downfield from the signal of the TMS (0 ppm). The peak assignment for protons in cyclopropyl, *cis* unsaturations, *trans* unsaturations and α-branched, β-hydroxyl systems were based on comparison of the spectra obtained with prior published spectra [19], [20], [21], [22].

## Results

### Ultrastructure of Cell Envelope

**Figure 1:**
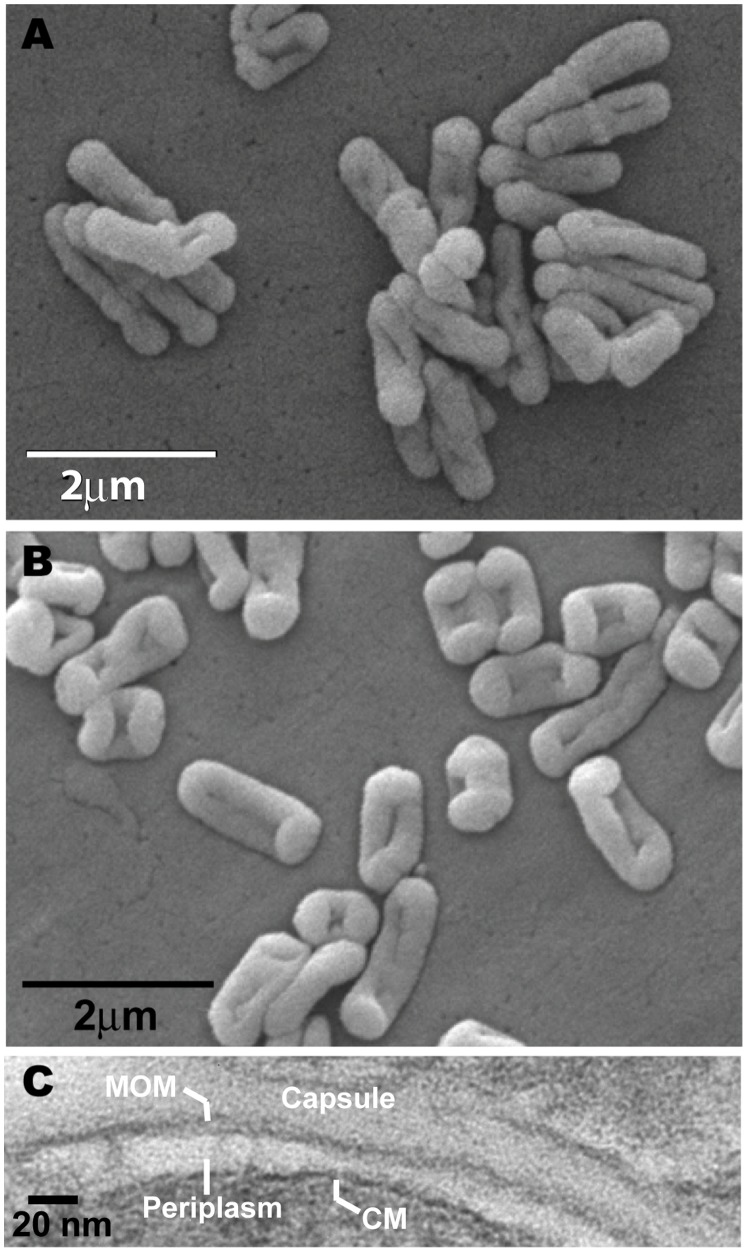
Electron micrographs. Scanning electron micrographs of *S. rotundus* (A) and *S. rugosus* (B)**.** (C) Transmission electron micrograph of *S. rugosus*. The bands are labeled as indicated. MOM stands for mycolate outer membrane and CM stands for cytoplasmic membrane.

*S. rotundus* and *S. rugosus* show differing smooth and rough colony forms, and display somewhat distinct appearance by electron microscopy (Fig. 1) [10]. Transmission electron microscopy (TEM) identified two layers showing the characteristic appearance of formed lipid membranes: triple bands composed of alternating electron dense and light regions. The ultrathin TEM images for *Segniliparus rugosus* are interpreted as an electron transparent periplasmic space, enclosed by a triple-layer of alternating dense and transparent bands typical of a mycolate membrane and surrounded by a less electron dense outermost capsule layer. The capsular layer was irregularly shaped and was present in *S. rugosus* but not in *S. rotundus*. The multilayered appearance of the *Segniliparus* envelope recapitulated most known aspects of mycobacterial structures.

### Segniliparus Mycolic Acid Classes are Defined by Chain Length

**Figure 2:**
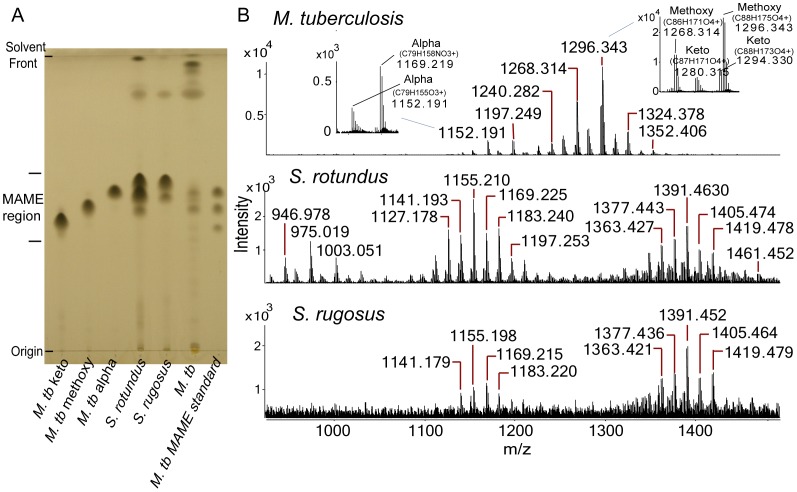
Purification and analysis of *Segniliparus* mycolates. (A) Normal phase one-dimensional thin-layer chromatography (1D TLC) of saponified and esterified lipids from *M. tuberculosis (M. tb)*, *S. rugosus* and *S. rotundus*. From left to right: Lanes 1, 2, 3, and 7 were loaded with purified keto (1), methoxy (2), alpha (3), or mixed (7) MAMEs of *M. tuberculosi*s. Lanes 4, 5, 6 were loaded with methylated saponificates containing both fatty acyl methyl esters and MAMEs derived from *S. rotundus* (4), *S. rugosus* (5), or *M. tuberculosis* (*M. tb*, 6). (B) Positive-ion mode Q-Tof ESI-MS analysis of total MAMEs derived from *M. tuberculosis* (top panels), with insets showing mass values that deduce to chemical formulas having either 3 (α) or 4 (keto, methoxy) oxygen atoms and compared with MAMEs from *S. rotundus* and *S. rugosus* (middle and bottom panels).

We methylated the free carboxyl groups of saponified mycolic acids and separated the individual mycolic acid methyl ester (MAME) classes using normal phase, one-dimensional thin layer chromatography (1D TLC). As expected, *M. tuberculosis* MAMEs resolved into three spots, which comigrated with commercial standards for keto-, methoxy- or α-mycolates, which contain keto, methoxy or no oxygen-containing functional groups on the meromycolate chains (Fig. 2a, left). *M. tuberculosis* produced ions corresponding to the expected mass of protonated adducts of MAME with keto (*m/z* 1280.315) or methoxy (*m/z* 1268.314) functional groups, as well as ammoniated α-mycolates (*m/z* 1169.219) (Fig. 2b, inset). These molecules of known structure provided standards for comparison with unknown forms of mycolic acids.

For *Segniliparus rotundus*, we detected three spots, but these did not precisely co-migrate with mycobacterial mycolates. For *S. rugosus*, we consistently detected two spots. To determine the chemical basis for TLC separation of *Segniliparus* MAMEs into two or three subclasses, we carried out positive-ion mode LC-ESI-MS with high accuracy TOF detection (Fig. 2b, Fig. S1 and S2). *S. rotundus* and *S. rugosus* produced mycolic acids varying over a much wider mass range, as compared to *M. tuberculosis.* This difference in molecular size was particularly apparent for *S. rotundus,* which were detected from *m/z* 946.978 to 1461.452, which was more than twice the mass variance for individual molecular species of MAMEs observed in *M. tuberculosis*, which spanned from *m/z* 1152.191 to 1352.406 (Fig. 2b). Further, the overall ion profiles showed MAMEs from *S. rotundus* were segregated into three groups, comprised of three alkane series that differed in the deduced number of carbon atoms, whereas *S. rugosus* forms only two such groups. These overall patterns suggested that the three nearly separate bands in *S. rotundus* likely correspond to long, medium and short chain mycolates, whereas *S. rugosus* produced two spots, likely corresponding to medium and long chain MAMEs.

To test this hypothesis directly, each band was scraped and analyzed by preparative TLC and mass spectrometry. For *S. rotundus*, the most intense ions derived from the low, middle and high migrating bands were detected at *m/z* 975.021, 1127.175 and 1391.453, respectively (Fig. S3). It is notable that the middle and upper spots on TLC appear as a doublet and share ions in the intermediate mass range (Fig. S3). For *S. rugosus*, the middle and high bands showed distinct mass profiles with the most intense ions observed at *m/z* 1141.192 and 1363.424, respectively (Fig. S4). Thus, preparative TLC with mass spectrometry analysis confirmed that the three discretely eluting classes of *Segniliparus rotundus* and two classes of *S. rugosus* MAMEs represent three and two chain length variants respectively. Thus, mycobacteria and segniliparus species have different organizing principles of mycolate class structure, with the former based on oxygen substitutions and the latter based on the carbon chain length. This general hypothesis was further tested by detailed analyses of individual MAMEs in each subclass.

### Chemical Composition of 65 *Segniliparus* Homologous Series Mycolates

**Figure 3:**
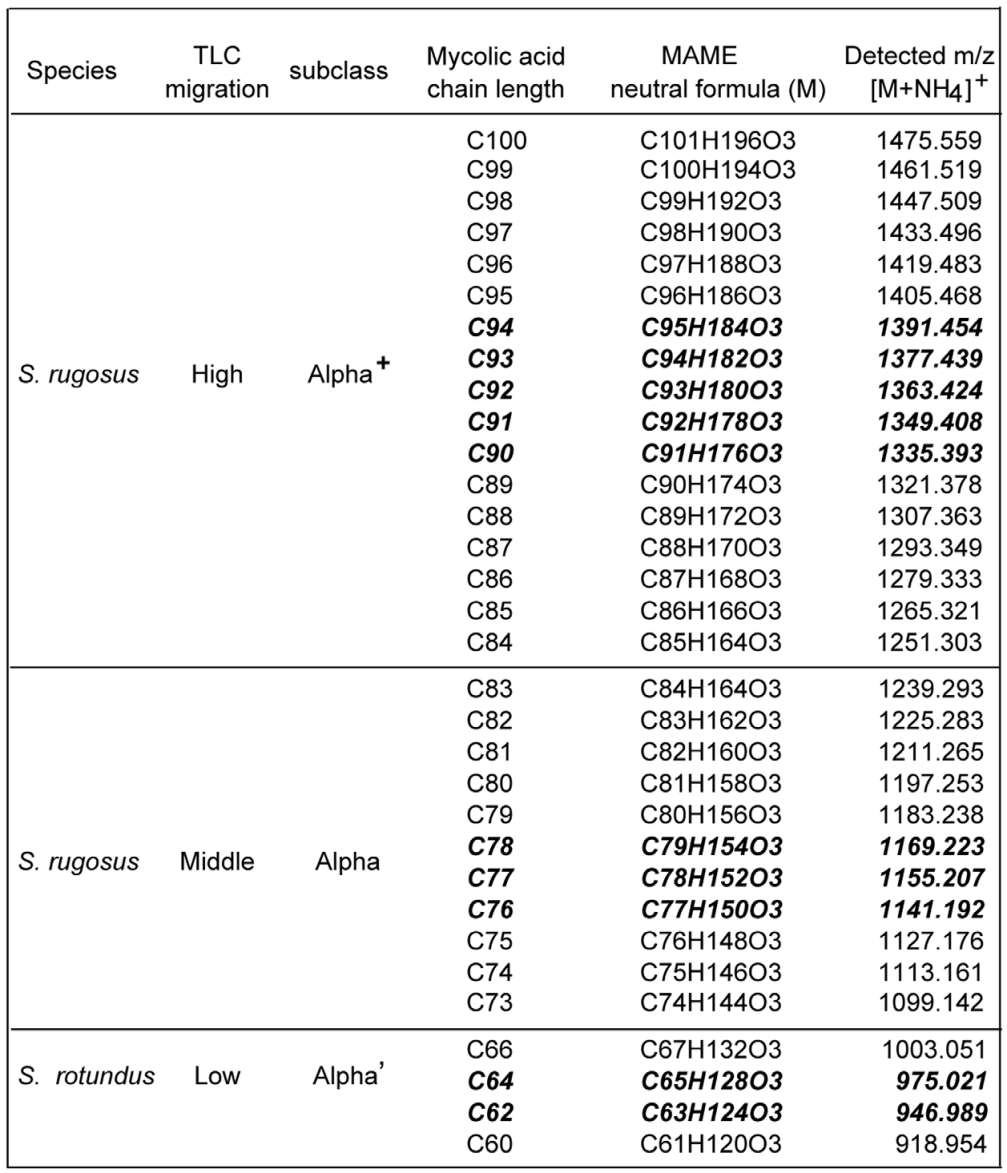
α^+^, α, and α’ mycolates. Positive-ion mode Q-Tof mass spectrometry of 1D TLC purified MAMEs derived from *S. rugosus* (high and middle bands) or *S. rotundus* (low band). The high migrating MAMEs with longer chain length, the middle migrating MAMEs with medium chain length, or the low migration MAMEs with shorter chain length were initially designated as α^+^, α, or α’ mycolate subclasses, respectively. The most intense molecular ions, observed here as ammonium adducts, present in each subclass are indicated in bold and italics.

For α-mycolates, the general chemical structure is deduced to give an [M+H]^+^ ion corresponding to O(3), C(X+1), H[2(X+1-Y)+1], where X is the number of carbons and Y is the number of unsaturations and cyclopropyl groups. Ammoniated adducts correspond to O(3), N(1), C(X+1), H[2(X+1-Y)+4]. For example, *S. rotundus* ions detected at *m/z* 1374.427 and 1391.454 were deduced as C_95_H_185_O_3_ and C_95_H_188_NO_3_, respectively (Figs. S2 and S1). The former is a proton adduct, while the later is an ammonium adduct, with both molecules having a chain length of C94 (X = 94) with three unsaturations (Y = 3). The mass error for both ions is 0.005 atomic mass units (amu) compared with the calculated *m/z* 1374.432 and 1391.459. Furthermore, MAME with oxygenated R groups deviate from this prediction in defined ways, with the key prediction that four, rather than three oxygen atoms are present. Systemic application of these rules to all ions detected by high accuracy measurements from the TOF detector to the 62 molecular ions for *S. rotundus* and 46 ions in *S. rugosus*, which allowed assignment of the chemical composition of 37 molecular forms of *S. rotundus* and 28 molecular species of *S. rugosus*. The molecular species are summarized in Fig. 3 and ions are comprehensively detailed in Figs. S1 and S2. The chemical composition assignments were considered reliable because ions were detected within narrow ranges of error expected for the method, except for compounds at the outer ranges of the chain length spectrum, which produced trace signals for which larger mass errors are expected. Further evidence for the chemical assignments included the relative intensity of isotopes, which matched those of high carbon content molecules and received high scores for isotope matching (not shown). Further, most deduced neutral structures [M], were detected both as protonated and ammoniated species (Figs. S2 and S1).

The chemical compositions of these 65 molecules confirmed that the chemical features, which organize *Segniliparus* mycolates into 3 alpha mycolic acid subclasses. In all cases the deduced chemical formula for *Segniliparus* mycolates contains three oxygen atoms. Assuming that these are carried on the carboxyl ester and the β hydroxyl group, no oxygen-containing R groups are present on the meromycolate chains. The alpha mycolic acids separated into α^+^-mycolates (C84–C100, *m/z* 1251.302 to 1475.518), α-mycolates (C72–C84, *m/z* 1085.130 to 1253.305) and short α’-mycolates (C58–C70, *m/z* 890.920 to 1059.109). The average length of *Segniliparus* α-mycolates (C94) greatly exceeded those lengths of mycolates in *M. tuberculosis* (C79), which are normally known as long chain mycolates (Fig. 2b), leading to their designation as ultralong mycolates. The C100 mycolate described here is to our knowledge the longest known natural mycolic acid.

### Alpha Chain Length Analysis

**Figure 4:**
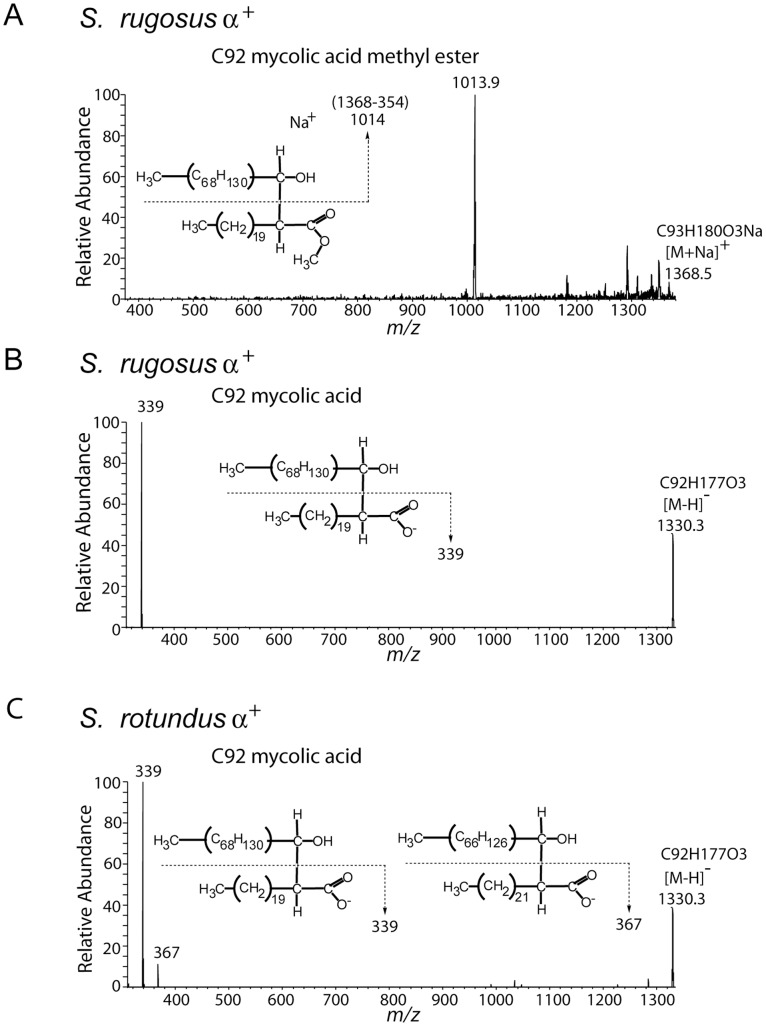
Collision induced dissociation mass spectrometry demonstrates alpha branch chain length. (A) Purified *S. rugosus* MAMEs found in the high migrating fraction on the TLC plate were dissolved in chloroform and methanol solution, loaded into a nanospray tip and analyzed by positive-ion mode electrospray ionization mass spectrometry with ion trapping. The ion at *m/z* 1368.5 [M+Na]^+^ corresponding to C92 mycolic acid methyl ester was subjected to CID-MS, yielding a fragment ion corresponding to a sodium adduct of the meroaldehyde chain (*m/z* 1013.9) which indicates a neutral loss of the α-chain-derived C22∶0 methyl ester. (B–C) Bacterial pellets of *S. rugosus* and *S. rotundus* were extracted with chloroform, diluted with methanol and analyzed by negative-ion mode electrospray ionization mass spectrometry. Both samples contain an ion at m/z 1330.3 ([M-H]^−^) corresponding to C92 mycolic acid, which was subjected to CID-MS analysis. The product ion 339 from *S. rugosus* (B) and ions 339 and 367 from *S. rotundus* (C) indicate the presence of C22∶0 and C24∶0 α-branch-derived fatty acids.

To confirm the length of the alpha branch, we carried out nanospray MS with collision-induced dissociation (CID) analysis (Fig. 4). In the positive-ion mode analysis, we found that ions corresponding to sodium adducts [M+Na]^+^ of C92 and C94 MAMEs from the *S. rugosus* α^+^ fraction, as well as C78 MAMEs, each generated a fragment of *m/z* 354 (Fig. 4a and data not shown). This fragment is attributed to a C22∶0 fatty methyl ester, which was cleaved at the C2–C3 position [22]. Because the product ions, including the meroaldehyde chain, are not always stable in the positive-ion mode, we sought to improve sensitivity by carrying out similar experiments on free mycolic acids (MA) in the negative ion mode [23], [24]. In both species, we isolated ions at *m/z* 1386 (C96 MA), 1330 (C92 MA), and 1136 (C78 MA), which were deduced as [M-H]^−^ of C96 MA, C92 MA and C78 MA. Additionally, for *S. rotundus*, we detected *m/z* 969, which was deduced as C66 MA. Each of these ions was subjected to CID-MS analysis. *S. rugosus* mycolates all generated single product ion 339 and no detectable chain length series (Fig. 4b). In contrast, ions 339 and 367 were found in *S. rotundus* (Fig. 4c). We interpret these patterns as α-chain derived fatty acyl chains of C22∶0 in *S. rugosus,* but C22∶0 and C24∶0 in *S.rotundus*. These results are in agreement with a prior pyrolysis analysis [10].

### Nuclear Magnetic Resonance Analysis

**Figure 5:**
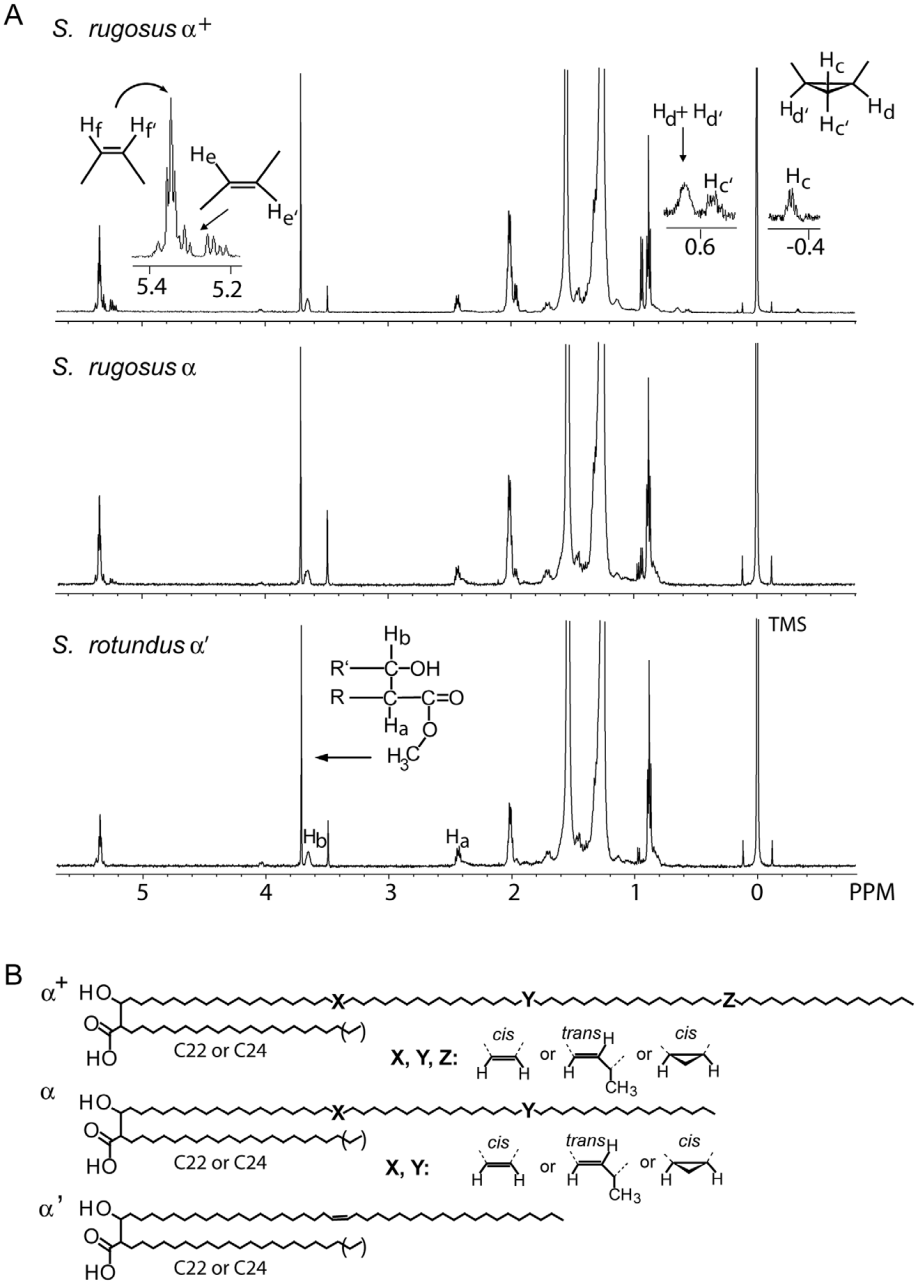
1H NMR analysis and structural summary of segnilomycolates. (A) 1D TLC purified MAMEs were analyzed by 1H NMR. Spectral assignment for the chemical shifts and splitting patterns of mycolates were based on comparison with values reported in the literature. The large peak at 1.56 ppm was attributed to water and the singlet at 3.49 ppm was attributed to methanol in the samples. (B) Structural summary of *Segniliparus* mycolic acids. The number of carbons between the functional groups on the meromycolate chains were not determined here, but are based on approximate lengths previously reported.

These initial conclusions, based on mass measurements, were further tested by 1H-NMR analysis of each class. For the high migrating *S. rotundus* doublet, there is overlap in chain length of the α^+^ and α mycolates. Therefore, these two samples are considered enriched for homogenous chain length, but are not pure class-specific mixtures (Fig. S3). Therefore, most 1H-NMR analyses was carried out on purified preparations of *S. rugosus* α^+^ and α-mycolates, as well as *S. rotundus* α’-mycolates (Fig. 5a). The proton resonances of mycolates were assigned based on published studies [19], [20], [21], [22]. Strong signals corresponding to methylene (CH_2_) protons in the mycolate chains were seen at 1.2–1.5 ppm, and the terminal methyl (CH_3_) protons appeared at 0.88 ppm. Characteristic protons of the Cα branched (H_a_, 2.43 ppm, 1 H, m), the hydroxylated Cβ (H_b_, 3.65 ppm, 1 H, m), and the terminal methyl group in ester linkage to the mycolate (3.71 ppm, 3 H, s) were identified in all five lipid samples, confirming the presence of the α-branch, β-hydroxyl and carboxylate, which define these lipids as mycolic acids.

The presence, number and stereochemistry of oxygen-containing R groups, unsaturations or cyclopropyl groups in the meromycolate chain are potentially important because these deviations from otherwise straight alkane structure influence the packing and thereby affect the fluidity of the mycolate membrane [3], [25]. We determined the number and orientation of the functional groups in these purified α-mycolates by the ^1^H-NMR chemical shifts and the integration of proton signals. As predicted by mass spectrometry, signals for methoxy-mycolate protons (3.3 ppm, 3 H) and for keto-mycolate, protons of CH_2_ (∼2.4 ppm, 2 H) and CH (∼2.5 ppm, 1 H), were absent from all 5 preparations of *Segniliparus* MAMEs, confirming the lack of oxygenated structures in the meromycolate chain.

In all purified mycolates, characteristic olefinic protons were identified. The signals for *cis*-double bond protons (H_f_, H_f_’) were at 5.35 ppm and *trans*-double bond protons (H_e_, H_e_’) were at 5.31 and 5.22 ppm. Methylene protons adjacent to the double bonds were located at 2.0 ppm and 1.98 ppm for *cis* and *trans*, respectively (Fig. 5a, S5). The signals for branched methyl groups adjacent to a *trans* double bond resonated at 0.94 ppm. In addition to simple unsaturations, the signals at −0.34, 0.55 and 0.64 ppm, attributed to the protons on the *cis* di-substituted cyclopropane ring, were detected in ultralong α^+^ mycolates of both strains and α mycolates of *S. rotundus* (Fig. 5a top panel and Fig. S5). The 1H-NMR results clearly indicated that *Sengiliparus* mycolates contain only double bonds and cyclopropanation as functional groups in their meromycolate chains.

The relationship of olefinic protons, two for each double bond, and a total of four protons on the Cβ (3.65 ppm) and methyl group (3.71 ppm), provide the basis for integration to establish the number and configuration of double bonds in each class (Fig. 5a). Based on integration of proton signals, *S*. *rugosus* α^+^ mycolates contain 3 unsaturations, which are deduced to be 60 percent *cis* and 30 percent *trans* double bonds, with trace levels of *cis* cyclopropanation. *S. rugosus* α mycolates contain 2 unsaturations, which are deduced to be comprised of 80 percent *cis* and 20 percent *trans* double bonds. *S. rotundus* α’ mycolates contain one double bond, which gives signals corresponding to the *cis*-orientation only. NMR signals for *S*. *rotundus* α^+^ and α preparations independently support these conclusions, but cannot be interpreted quantitatively due to the enriched, rather than pure nature of these preparations (Figs. S3 and S5).

### Integration of MS and NMR Results

Mycolates are synthesized as a homologous series of molecules that differ by C_2_H_4_ in length. *Trans-* but not *cis-*double bonds introduce an extra methyl branch, which contributes to C_3_H_6_. Therefore, mixed MAMEs comprised of molecules possessing and lacking *trans* unsaturations or *cis*-cyclopropanations typically appear as two overlapping alkane series in which each molecule differs by CH_2_. For the molecules only containing double bonds, these rules predict that odd chain mycolates are seen only in *trans* unsaturated molecules. Cross comparison of mass spectra (Fig. S3, S4) and NMR (Fig. 5a) indicate that the ratio of even to odd carbon numbers correlates with the percentage of *cis* unsaturated to *trans* unsaturated. For example, NMR of *S. rotundus* α’ mycolates indicates pure *cis* mycolates and is observed as a single series of molecules that differ by C_2_H_4_ from one another. Mixed *cis* and *trans* mycolates from *S. rugosus* α and α^+^ classes showed a minor series of odd-numbered mycolates in which the intensity of the minor odd chain species correlates with the predicted *trans* unsaturations from NMR data. The combined results of the mass spectrometry and NMR analyses identify chain length as the key feature, which separates these 3 classes and support the assignment of meromycolate structures within each class, as summarized in Fig. 5b.

## Discussion

This study identifies naturally occurring C100 lipids, whose long chain length represents a biochemical extreme that informs some general controversies related to chemotaxonomy and mycolate membrane structure. Despite genetic clustering with corynebacteria based on 16S RNA, the identification of long chain C58–C100 mycolic acids provide clear evidence for chemotaxonomic divergence of these two *Segniliparus* species from corynebacteria, which elaborate outer membranes with mycolates in the range of C22–36. *Segniliparus* mycolates are also distinguished from those in *M. tuberculosis* based on longer chain length and lack of oxygen-containing R groups. Thus, the chemical data presented here add to evidence that *Segniliparus* is unique and appropriately assigned to a new genus.

The term segnilomycolates is suggested for this class of molecules, whose distinguishing features include lack of oxygen-containing substitutions on the meromycolate chains, high degree of *cis* unsaturation, ultralong chain length and the large range of chain length present in a single bacterial culture. The variance in chain length of individual molecular species, which presumably form into one mycolate membrane, is unprecedently large. For example, mycolates reported here are both shorter and longer than those from *Mycobacteria* (C60–90) and *Tsukamurella* (C64–78). The observed variance is also distinctly atypical for any bacterium in the suborder *Corynebacterineae*. More typically, mycolyl lipids vary by 8 to 20 methylene groups, rather than 42 as seen here. Thus, intraorganismal variation in chain length raises new questions about the native conformations of mycolic acids.

The precise role for such a structural diversity of *Segniliparus* α-mycolic acids is unknown, but likely influences mycolate membrane fluidity and permeability. Biochemical and structural data show that the unusual mycolates observed here form a mycolate membrane that is analogous to those found among other actinobacteria. For example, previous studies of *S. rugosus* and *S. rotundus* revealed brightly staining acid fast cells [10], a biochemical feature of formed mycolate membranes. Further, we provide electron micrographic evidence of an intact membrane composed of three layers of alternating electron dense and electron transparent layers. Thus, segnilomycolates likely do form a membrane and that it is not radically different in its ultrastructure compared to mycobacteria. These observations invite consideration of how the unique chemical properties of segnilomycolates might influence lipid membrane dynamics.

Detecting the long chain length and lack of oxygenated meromycolate chains in combination was initially surprising because both modifications, acting separately, would be expected to reduce membrane transition temperatures. These observations invite countervailing chemical modifications that might promote fluidity. Subsequently, we observed high degrees of *cis* unsaturation, which bend meromycolate chains and have the largest effects to raise transition temperatures, which might represent a natural modification to mitigate overly tight packing [25]. A simple model to accommodate longer chain length would be to produce a larger trans-membrane span. However, this simple model does not account for the C42 lipid length disparity within one organism. Further, progressively longer α’, α, and α^+^ mycolates are associated with one, two or three unsaturations or cyclopropyl groups, respectively (Fig. 5b). Relatively new models of mycolate packing emphasize a possible “w” fold whereby typical mycolic acid meromycolate chains in the range of C80 use two functional groups to fold into three sections, which combined with the α-chain, appears like a “w” [26], [27].

Combining these two ideas, these patterns suggest that longer chain length is associated with more highly folded molecules, rather than outstretched molecules that would span a larger transverse distance across the membrane. This mechanism might account for a homogenously thick membrane composed of mycolic acids of such divergent length. This mechanism is in agreement with prior observations of mycolic acid membrane width and density patterns, as well as simulations that predict mycolic acid folding [27], [28]. Current models predict that the α-chain and three segments of mycobacterial meromycolate chains, which are approximately C68, fold at two points of unsaturation or cyclopropanation, to create a “w”. Therefore, our data extend existing models to predict that the α^+^ mycolates use their α-branch, plus triply substituted, quadruply folded meromycolate chains to yield a “w +1” configuration. By this same logic, singly unsaturated α’ mycolates might represent “N” folding pattern. Future studies of membrane fluidity and in situ membrane dynamics will test these models. Last, the unusual structures seen here support further work on the role of *Segniliparus* type II fatty acyl synthases and CMAS genes to better understand enzyme factors that control chain length and unsaturation [3], [4], [5].

## Supporting Information

Figure S1 Elemental composition of *S. rotundus* and *S. rugosus* mycolates derived from ammoniated adducts. MAMEs derived from *S. rotundus* and *S. rugosus* were analyzed by positive-ion mode Q-Tof mass spectrometry. The empiric formulas were deduced from the accurate mass of MAME ammonium adducts. The difference between the detected and the calculated *m/z* is reported as the error.
(TIF)

Figure S2 Elemental composition of *S. rotundus* and *S. rugosus* mycolates derived from protonated adducts. MAMEs derived from *S. rotundus* and *S. rugosus* were analyzed by positive-ion mode Q-Tof mass spectrometry. The empiric formulas were deduced from the accurate mass of MAME proton adducts. The difference between the detected and the calculated *m/z* is reported as the error.
(TIF)

Figure S3 Positive-ion mode of Q-Tof mass spectrometry of MAMEs derived from *Segniliparus rotundus* that were separated and recovered from 1D TLC as high, middle or low bands, as illustrated in Fig. 2a. Due to lack of complete separation of the middle and high migrating doublet observed on TLC, the high migrating band contains ions also seen in the middle migrating band. Only ammonium adducts were labeled for high and middle bands. The ammonium or proton adducts detected in the low migrating band were labeled as indicated.
(TIF)

Figure S4 Positive-ion mode Q-Tof mass spectrometry of MAMEs derived from *Segniliparus rugosus* were separated and recovered from 1D TLC as high or middle bands as illustrated in Fig. 2a. Only ammonium adducts were labeled.
(TIF)

Figure S5 *S. rotundus* MAMEs isolated from the 1D TLC high migrating band and middle migrating band were analyzed by 1H NMR spectroscopy. As indicated in Fig. S3, these are enriched for chain length-based classes, but are not fully purified based on chain length. However, key aspects of the spectra are recapitulated in the more fully purifiable compounds shown in Fig. 5.
(TIF)

## References

[1] Alonso-Gutiérrez J, Figueras A, Albaigés J, Jiménez N, Viñas M (2009). Bacterial communities from shoreline environments (Costa da Morte, Northwestern Spain) affected by the prestige oil spill.. Appl Environ Microbiol.

[2] Tlaskalova-Hogenová H, Stepánková R, Hudcovic T, Tucková L, Cukrowska B (2004). Commensal bacteria (normal microflora), mucosal immunity and chronic inflammatory and autoimmune diseases.. Immunol Lett.

[3] Barry CE, Lee RE, Mdluli K, Sampson AE, Schroeder BG, Slayden RA, Yuan Y (1998). Mycolic acid: structure, biosynthesis and physiological functions (review).. Prog Lipid Res.

[4] Marrakchi H, Bardou F, Lanéelle MA, Daffé M, Daffé M, Reyrat JM (2008). A comprehensive overview of mycolic acid structure and biosynthesis..

[5] Asselineau C, Asselineau J, Lanéelle G, Lanéelle MA (2002). The biosynthesis of mycolic acids by Mycobacteria: current and alternative hypotheses.. Prog Lipid Res.

[6] Hoffmann C, Leis A, Niederweis M, Plitzko JM, Engelhardt H (2008). Disclosure of the mycobacterial outer membrane: cryo-electron tomography and vitreous sections reveal the lipid bilayer structure.. Proc Natl Acad Sci U S A.

[7] Barkan D, Liu Z, Sacchettini JC, Glickman MS (2009). Mycolic acid cyclopropanation is essential for viability, drug resistance, and cell wall integrity of *Mycobacterium tuberculosis*.. Chem Biol.

[8] Dao DN, Sweeney K, Hsu T, Gurcha SS, Nascimento IP (2008). Mycolic acid modification by the *mmaA4* gene of *M. tuberculosis* modulates IL-12 production.. PLoS Pathog.

[9] Bhatt A, Molle V, Besra GS, Jacobs WR, Kremer L (2007). The *Mycobacterium tuberculosis* FAS-II condensing enzymes: their role in mycolic acid biosynthesis, acid-fastness, pathogenesis and in future drug development.. Mol Microbiol.

[10] Butler WR, Floyd MM, Brown JM, Toney SR, Daneshvar MI (2005). Novel mycolic acid-containing bacteria in the family *Segniliparaceae* fam. nov., including the genus *Segniliparus* gen. nov., with descriptions of *Segniliparus rotundus* sp. nov. and *Segniliparus rugosus* sp. nov.. Int J Syst Evol Microbiol.

[11] Hansen T, Van-Kerckhof J, Jelfs P, Wainwright C, Ryan P (2009). *Segniliparus rugosus* infection, Australia.. Emerg Infect Dis.

[12] Butler WR, Sheils CA, Brown-Elliott BA, Charles N, Colin AA (2007). First isolations of *Segniliparus rugosus* from patients with cystic fibrosis.. J Clin Microbiol.

[13] Evans RH (2011). *Segniliparus rugosus*-associated bronchiolitis in California sea lion.. Emerg Infect Dis.

[14] Sommerville J, Scheer U (1987). Electron Microscopy in Molecular Biology: A Practical Approach; J. S, U. S, editors. Eynsham, Oxford. USA: Oxford University Press.. 264 p.

[15] Minnikin DE, Hancock IC, Poxton IR (1998). Isolation and purification of mycobacterial wall lipids..

[16] Goodfellow M, Collins MD, Minnikin DE (1976). Thin-layer chromatographic analysis of mycolic acid and other long-chain components in whole-organism methanolysates of coryneform and related taxa.. J Gen Microbiol.

[17] Minnikin DE, Hutchinson IG, Caldicott AB, Goodfellow M (1980). Thin-layer chromatography of methanolysates of mycolic acid-containing bacteria.. Journal of Chromatography A.

[18] Layre E, Sweet L, Hong S, Madigan CA, Desjardins D (2011). A comparative lipidomics platform for chemotaxonomic analysis of *Mycobacterium tuberculosis*.. Chem Biol.

[19] Quemard A, Lanéelle MA, Marrakchi H, Promé D, Dubnau E (1997). Structure of a hydroxymycolic acid potentially involved in the synthesis of oxygenated mycolic acids of the *Mycobacterium tuberculosis* complex.. Eur J Biochem.

[20] Schroeder BG, Barry CE (2001). The specificity of methyl transferases involved in trans mycolic acid biosynthesis in *Mycobacterium tuberculosis* and *Mycobacterium smegmatis*.. Bioorg Chem.

[21] Watanabe M, Aoyagi Y, Ridell M, Minnikin DE (2001). Separation and characterization of individual mycolic acids in representative mycobacteria.. Microbiology.

[22] Mederos L, Valdivia JA, Valero-Guillén PL (2007). Analysis of the structure of mycolic acids of *Mycobacterium simiae* reveals a particular composition of α-mycolates in strain ‘habana’ TMC 5135, considered as immunogenic in tuberculosis and leprosy.. Microbiology.

[23] Shui G, Bendt AK, Pethe K, Dick T, Wenk MR (2007). Sensitive profiling of chemically diverse bioactive lipids.. J Lipid Res.

[24] Hsu FF, Soehl K, Turk J, Haas A (2011). Characterization of mycolic acids from the pathogen *Rhodococcus equi* by tandem mass spectrometry with electrospray ionization.. Anal Biochem.

[25] Liu J, Barry CE, Besra GS, Nikaido H (1996). Mycolic acid structure determines the fluidity of the mycobacterial cell wall.. J Biol Chem.

[26] Villeneuve M, Kawai M, Kanashima H, Watanabe M, Minnikin DE (2005). Temperature dependence of the Langmuir monolayer packing of mycolic acids from *Mycobacterium tuberculosis*.. Biochim Biophys Acta.

[27] Villeneuve M, Kawai M, Watanabe M, Aoyagi Y, Hitotsuyanagi Y (2007). Conformational behavior of oxygenated mycobacterial mycolic acids from *Mycobacterium bovis* BCG.. Biochim Biophys Acta.

[28] Zuber B, Chami M, Houssin C, Dubochet J, Griffiths G (2008). Direct visualization of the outer membrane of mycobacteria and corynebacteria in their native state.. J Bacteriol.

