# Supplementary figures and images for: Ultralong C100 Mycolic Acids Support the Assignment of Segniliparus as a New Bacterial Genus
Source: PLoS One. 2012 Jun 14;7(6):e39017. doi: 10.1371/journal.pone.0039017 (PMC3375245; doi:10.1371/journal.pone.0039017)

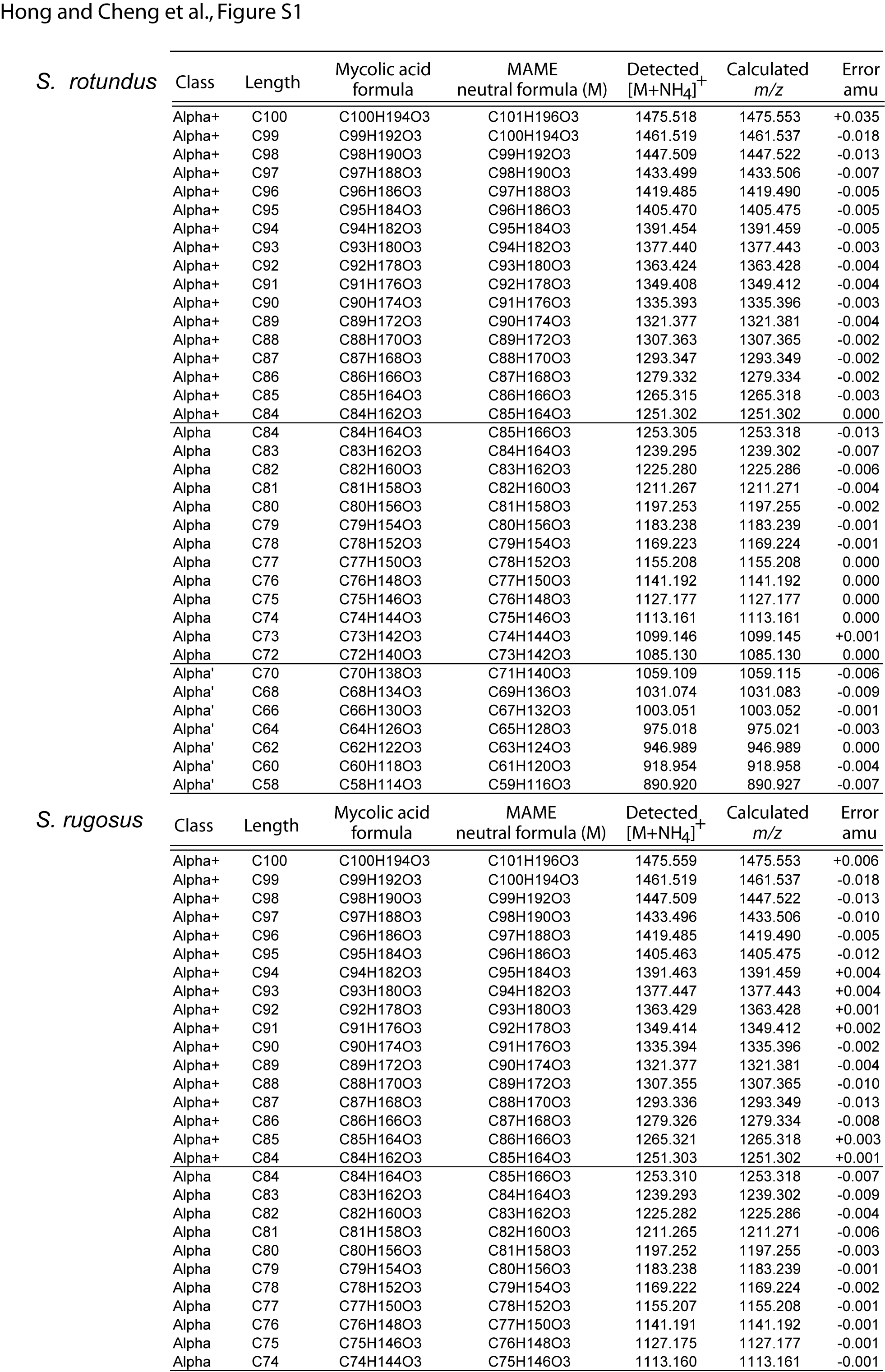

Supplement: Figure S1 — Elemental composition of S. rotundus and S. rugosus mycolates derived from ammoniated adducts. MAMEs derived from S. rotundus and S. rugosus were analyzed by positive-ion mode Q-Tof mass spectrometry. The empiric formulas were deduced from the accurate mass of MAME ammonium adducts. The difference between the detected and the calculated m/z is reported as the error. (TIF) [file pone.0039017.s001.tif]

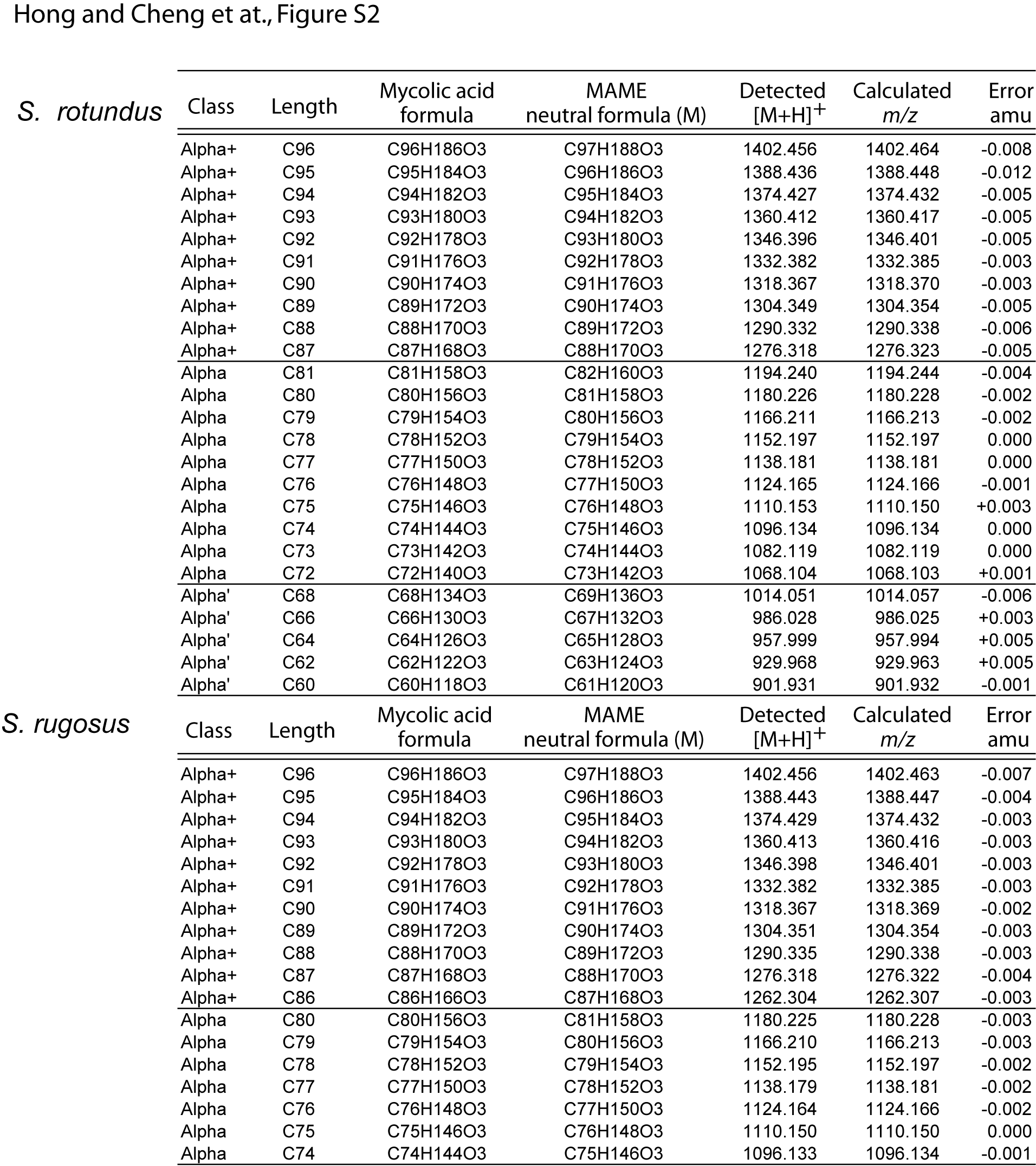

Supplement: Figure S2 — Elemental composition of S. rotundus and S. rugosus mycolates derived from protonated adducts. MAMEs derived from S. rotundus and S. rugosus were analyzed by positive-ion mode Q-Tof mass spectrometry. The empiric formulas were deduced from the accurate mass of MAME proton adducts. The difference between the detected and the calculated m/z is reported as the error. (TIF) [file pone.0039017.s002.tif]

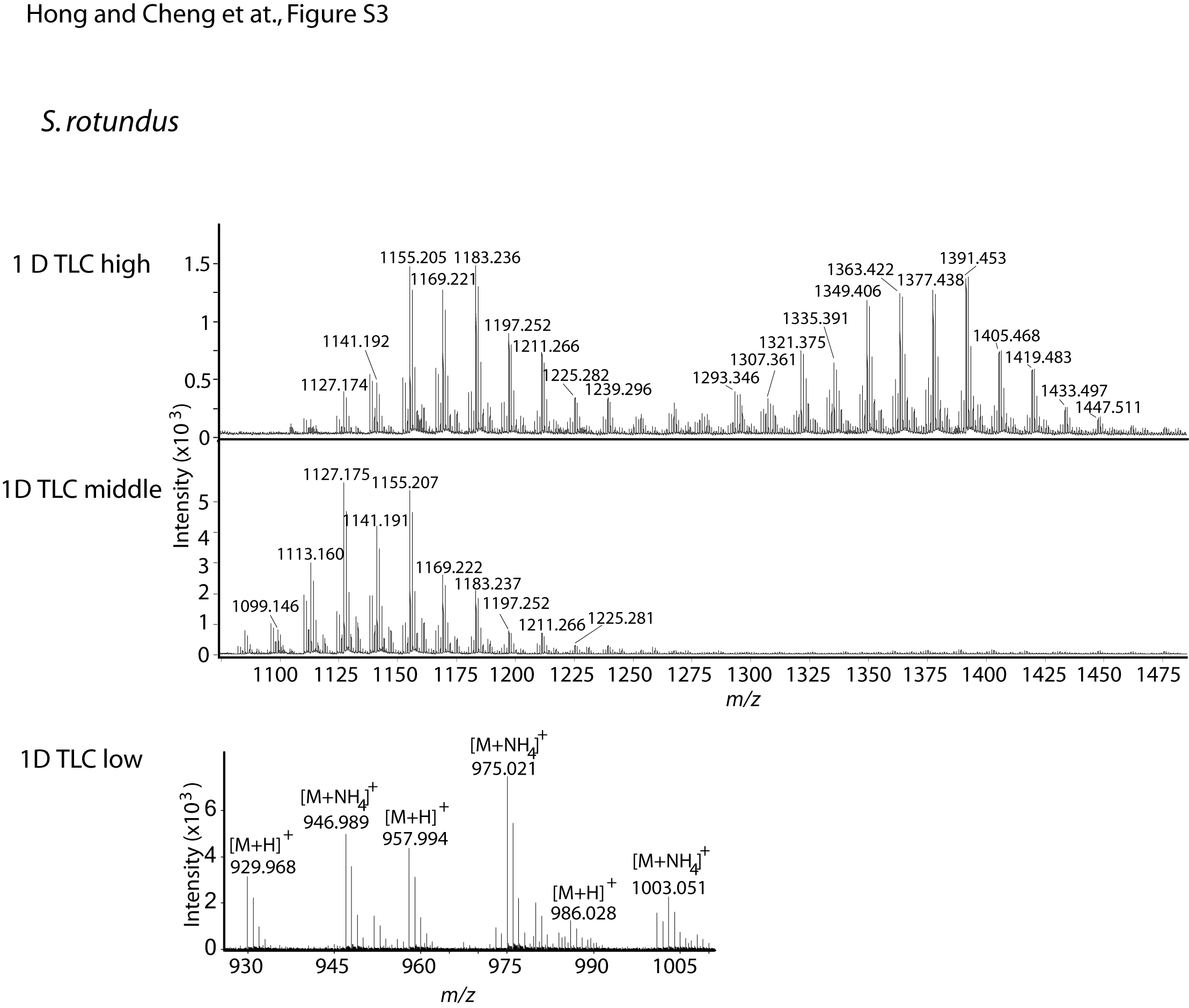

Supplement: Figure S3 — Positive-ion mode of Q-Tof mass spectrometry of MAMEs derived from Segniliparus rotundus that were separated and recovered from 1D TLC as high, middle or low bands, as illustrated in Fig. 2a. Due to lack of complete separation of the middle and high migrating doublet observed on TLC, the high migrating band contains ions also seen in the middle migrating band. Only ammonium adducts were labeled for high and middle bands. The ammonium or proton adducts detected in the low migrating band were labeled as indicated. (TIF) [file pone.0039017.s003.tif]

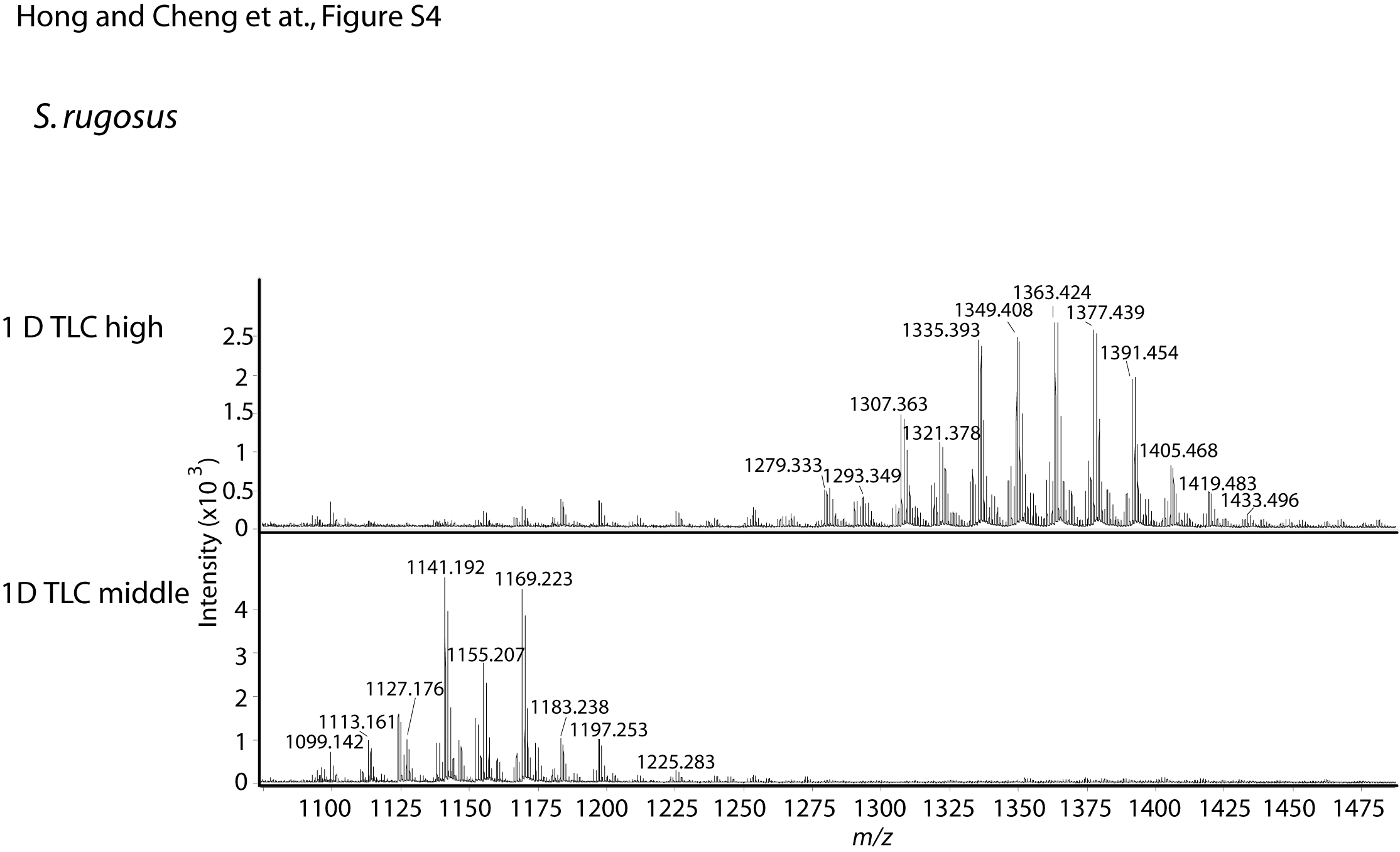

Supplement: Figure S4 — Positive-ion mode Q-Tof mass spectrometry of MAMEs derived from Segniliparus rugosus were separated and recovered from 1D TLC as high or middle bands as illustrated in Fig. 2a. Only ammonium adducts were labeled. (TIF) [file pone.0039017.s004.tif]

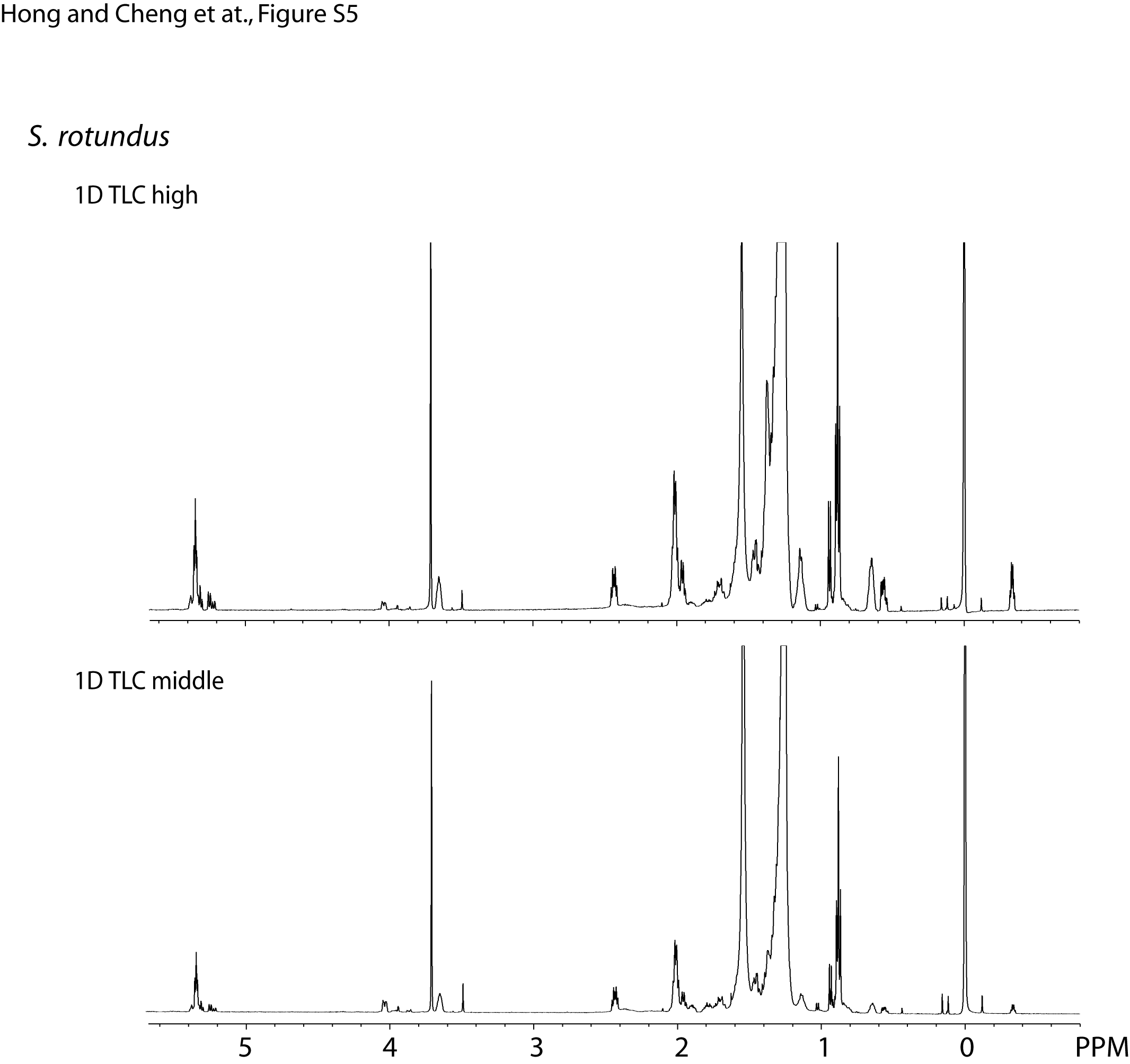

Supplement: Figure S5 — S. rotundus MAMEs isolated from the 1D TLC high migrating band and middle migrating band were analyzed by 1H NMR spectroscopy. As indicated in Fig. S3, these are enriched for chain length-based classes, but are not fully purified based on chain length. However, key aspects of the spectra are recapitulated in the more fully purifiable compounds shown in Fig. 5. (TIF) [file pone.0039017.s005.tif]
